# Chemical composition, antibacterial activity and action mechanism of different extracts from hawthorn (*Crataegus pinnatifida* Bge.)

**DOI:** 10.1038/s41598-020-65802-7

**Published:** 2020-06-01

**Authors:** Liang-Liang Zhang, Li-Fang Zhang, Jian-Guo Xu

**Affiliations:** 1 School of Chemistry and Material Science, Shanxi Normal University, Linfen, 041004 China; 2 School of Food Sciences, Shanxi Normal University, Linfen, 041004 China

**Keywords:** Biochemistry, Biological techniques, Biotechnology, Microbiology

## Abstract

Present study was designed to compared the total flavonoids and polyphenols contents and antibacterial activity of hawthorn extracts with different polarities as well as the underlying antibacterial mechanisms. The results showed that among all hawthorn extracts, methanol and ethanol extracts (ME and EE) exhibited high levels of total flavonoids and polyphenols contents, followed by acetone, ethyl acetate, trichloromethane and petroleum ether extracts. ME exhibited the strongest antibacterial activity against tested bacteria, especially *Staphylococcus aureus* with a 1.25 μg/mL of the minimum inhibitory concentration (MIC) and minimum bactericide concentration (MBC). Further analysis revealed that the main phenolic compounds from ME were epicatechin (281.6 mg/100 g DW), procyanidin B2 (243.5 mg/100 g DW), chlorogenic acid (84.2 mg/100 g DW) and quercetin (78.4 mg/100 g DW). The action mechanism of ME against *S. aureus* could be ascribed to ME damaging cell wall and cell membrane integrity, inhibiting intracellular enzyme activity, increasing reactive oxygen species (ROS), also changing expression of associated genes and then inducing apoptosis of *S. aureus*. In addition, the antimicrobial activity of ME against *S. aureus* has also been demonstrated to be efficient in the food matrix (whole milk).

## Introduction

*Crataegus pinnatifida* Bge. (Rosaceae), informally called hawthorn tree, is a widely cultivated perennial woody plant in China^1^. Hawthorn has an extremely long edible history and also a well-known Chinese herbal medicine^2^. Hawthorn is rich in carbohydrate, organic acids, vitamins and mineral elements^3^, but also has a range of diverse biological functions including antioxidant, antibacterial effect and pharmacological effects on digestive, cardiovascular and endocrine systems, neuroprotective function and preventing obesity^4–7^. Polyphenols have been recognized as one of mainly bioactive compounds in hawthorn, and it contained more than 150 phenolic compounds including procyanidins (procyanidin B2, procyanidin B5 and procyanidin C1), flavonoids (epicatechin, hyperoside, quercetin, rutin and isoquercitrin), and triterpenoids acid (ursolic acid, corosolic acid, oleanolic acid and maslinic acid)^8^.

Traditionally, hawthorn fruits are consumed directly or processed into jams, jellies, wine, juice and various sweet foods^9^. Now, with the booming of functional and health care industry, hawthorn extracts as a kind of nutrient enhancer have been widely used^10,11^. Wen *et al*. studied phenolic contents and cellular antioxidant activity of 80% acetone extracts from three varieties of hawthorn^6^. Zhang *et al*. found the ethyl acetate extracts were only effective in inhibition of LDL oxidation among examined four different solvents^12^. Miao *et al*. studied the antioxidant activity, chemical compositions of hawthorn extracts obtained using different solvents, the results showed that the highest total flavonoids and total polyphenols was obtained from 80% acetone extract, but the deionised water extract has the best DPPH scavenge capacity and ferric reducing power^13^. Therefore, the biological activities and health effects of hawthorn extracts can be greatly influenced by many factors such as extraction methods, origins and varieties. Among all these factors, extraction solvents, especially the polarity nature of them, showed the greatly significant effects on its phytochemical compositions and bioactivities^12–14^.

Therefore, the aim of the present study was conducted to investigate the total polyphenols and flavonoids contents and antibacterial activities of six hawthorn extracts with decreasing polarity (methanol, ethanol, acetone, ethyl acetate, trichloromethane and petroleum ether), and to further evaluate the possible action mechanism responsible for the antibacterial activity against sensitive strains. In addition, the antibacterial activity of ME in whole milk was also evaluated.

## Results

### Total flavonoids and total polyphenols contents

**Figure 1:**
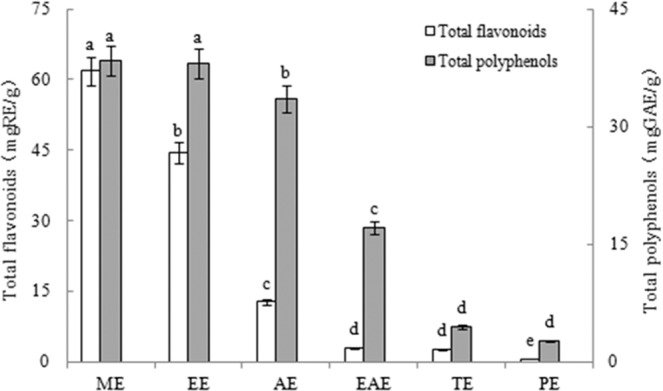
The content of total flavonoids and polyphenols of different extracts.

The contents of total flavonoids and polyphenols in six hawthorn extracts are presented in Fig. 1. The content of flavonoids in the different extracts showed a significant difference (*P* < 0.05) ranged from 0.59 to 61.84 mg RE/g DW. The highest total flavonoid content was obtained in ME, followed by ethanol (EE), acetone (AE), ethyl acetate (EAE), trichloromethane (TE) and petroleum ether (PE) extracts. The total polyphenols contents showed a significant difference (*P* < 0.05) and varied in the range from 2.66 to 38.40 mg GAE/g DW. Similar to total flavonoids, the total polyphenols contents decreased along with a decreasing order of solvent polarity (ME > EE > AE > EAE > TE > PE).

### Antibacterial activity

The DIZ values of the extracts from hawthorn are shown in Table 1. The results indicated that the ME, EE and AE had certain antibacterial activity on all of the tested food-borne pathogens, while EAE had antibacterial activity on *Staphylococcus aureus*, *Escherichia coli, Shigella dysenteriae* and *Salmonella typhimurium*. However, TE and PE had no significant antibacterial activity on all of the tested strains. Among them, ME and EE possessed the strongest antibacterial activity (20.4–29.7 mm).

**Table 1:** DIZ of hawthorn extracts against seven microorganisms.

Microorganisms	DIZ (mm)					
	ME	EE	AE	EAE	TE	PE
**Gram-positive**						
*S. aureus*	25.5 ± 1.6Ac	26.9 ± 0.7Aa	18.6 ± 0.2Bc	11.1 ± 0.8Cb	—	—
*B. subtilis*	22.7 ± 1.5Ad	24.1 ± 1.3Ab	17.2 ± 1.4Bc	—	—	—
*L. monocytogenes*	23.5 ± 0.9Ad	20.4 ± 0.8Bc	8.7 ± 2.6 Cd	—	—	—
**Gram-negative**					—	—
*E. coli*	29.7 ± 1.3Aa	27.1 ± 1.1Ba	20.5 ± 0.2Cb	14.7 ± 0.8 Da	—	—
*S. castellani*	31.7 ± 2.4Aa	23.8 ± 0.2Bb	22.4 ± 0.7Ba	13.8 ± 0.4 Da	—	—
*S. typhimurium*	27.7 ± 0.6Ab	25.8 ± 0.4Bab	18.9 ± 0.4 Cc	9.7 ± 0.5Dc	—	—
*P. aeruginosa*	26.2 ± 1.1Abc	26.9 ± 1.6Aa	18.1 ± 0.7Bc	—	—	—

Values represent means of three independent replicates ± SD. “−”: not detected. Different letters within row (upper case letters) and column (lower case letters) indicate statistically significant differences between the means (P < 0.05).

To further compared the antibacterial activities of different hawthorn extracts, the MIC and MBC values of the ME, EE and AE were compared (Table 2). ME exhibited stronger antibacterial activity than EE and EAE. Among all of tests, ME from hawthorn had a minimum MIC and MBC value of 1.25 μg/mL against *S. aureus* and *S. typhimurium*. Based on the results of MIC and MBC, ME was used for phenolic component analysis and investigating the mode of antibacterial action. Considering the common and serious pathogenic characteristics of *S. aureus* in the areas of food industry and safety, the antibacterial properties and mechanism of action of ME against *S. aureus* will be further investigated in this study.

**Table 2:** MIC (μg/mL) and MBC (μg/mL) of hawthorn extracts.

Microorganisms	ME		EE		AE	
	MIC	MBC	MIC	MBC	MIC	MBC
**Gram-positive**						
*S. aureus*	1.25	1.25	10	10	20	20
*B. subtilis*	10	20	20	20	40	80
*L. monocytogenes*	5	10	20	20	40	80
**Gram-negative**						
*E. coli*	10	20	20	20	40	40
*S. castellani*	10	20	10	20	10	40
*S. typhimurium*	1.25	1.25	10	10	10	10
*P. aeruginosa*	20	20	20	20	20	40

### Phenolic compositions of ME

Generally, not only the contents but also the compositions of total polyphenols had the impact on its bioactivities. Thus, the polyphenol composition of the ME from hawthorn were analyzed by HPLC. The chromatogram of reference standards and the ME from hawthorn at wavelength 280 nm are shown in Figure S1, and the analysis results are shown in Table 3. In the present study, eight phenolic compositions were determined and the most abundant two compounds were epicatechin and procyanidin B2 with their concentrations up to 281.6 and 243.5 mg/100 g DW, respectively. The levels of chlorogenic acid and quercetin were 84.2 and 78.4 mg/100 g DW. The other four compounds were catechin, paracoumaric acid, hyperoside and isoquercitrin and their contents were no more than 30 mg/100 g DW.

**Table 3:** The content of phenolic compounds identified from ME.

Peak No.	t_R_ (min)	Identification	content (mg/100 g DW)
1	20.3	catechin	27.1 ± 1.2
2	22.9	chlorogenic acid	84.2 ± 4.3
3	25	procyanidin B2	243.5 ± 12.6
4	28.6	epicatechin	281.6 ± 21.4
5	32.8	quercetin	78.4 ± 2.2
6	34.6	paracoumaric acid	24.9 ± 1.7
7	47.2	hyperoside	11.8 ± 0.5
8	48.9	isoquercitrin	9.6 ± 0.8

Values represent means of three independent replicates ± SD.

### Electron microscopic observations

**Figure 2:**
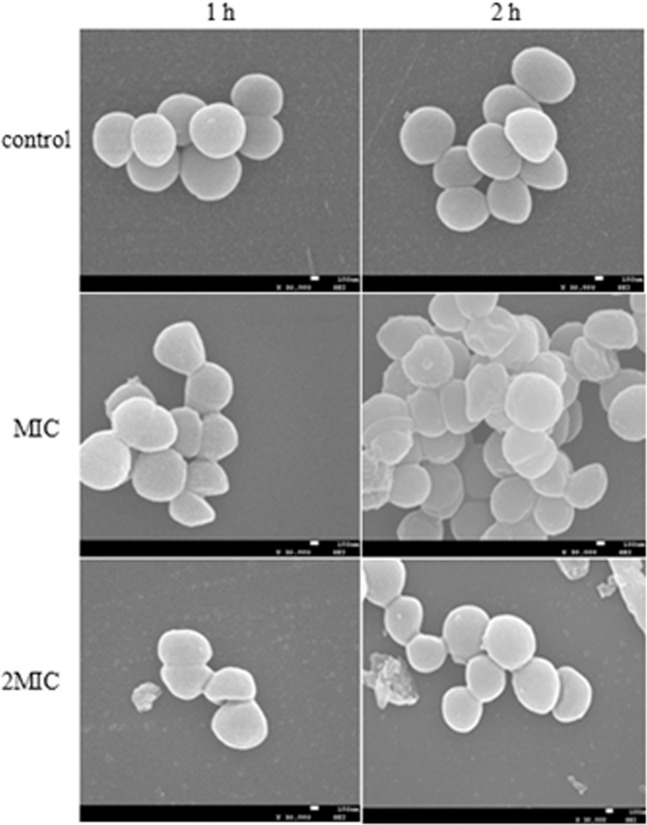
SEM of *S. aureus* untreated cells and after treatment with ME.

The influence of ME from hawthorn on the morphological and architecture of *S. aureus* was observed by SEM. According to the results, ME caused a significant change in the morphology of *S. aureus* (Fig. 2**)**. Compared to the untreated *S. aureus* with a typically spherical-shaped cell morphology and a relatively smooth surface, *S. aureus* treated with ME exhibited rough surface and had a lot of wrinkles without regular spherical-shaped, indicating that the cell wall was damaged. And the damage can get worse with the extension of treatment time and increase of concentration of ME.

### Cell membrane permeability

**Figure 3:**
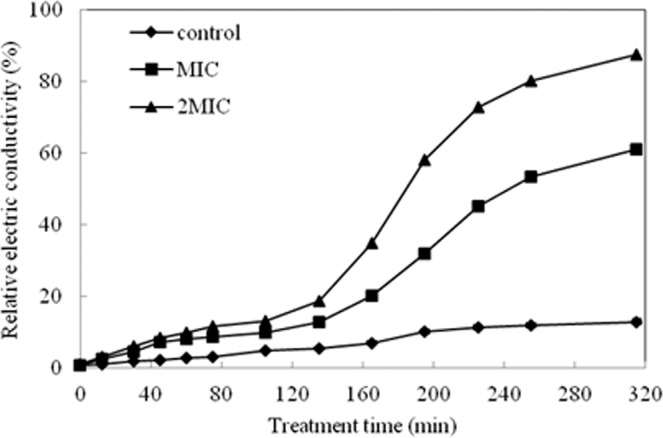
The effect of the ME on membrane permeability.

Figure 3 showed the effect of ME from hawthorn on the membrane permeability of *S. aureus* by electric conductivity assay. In the control group, there was little change in the relative electric conductivity during the whole experiment period. However, both MIC and 2MIC groups showed a significant increase in a dose-dependent manner after 2 h of ME treatment. These results indicated that ME treatment caused the leakage of intracellular electrolyte including K^+^, Ca^2+^, Na^+^ and so on.

### Cell membrane potential

**Figure 4:**
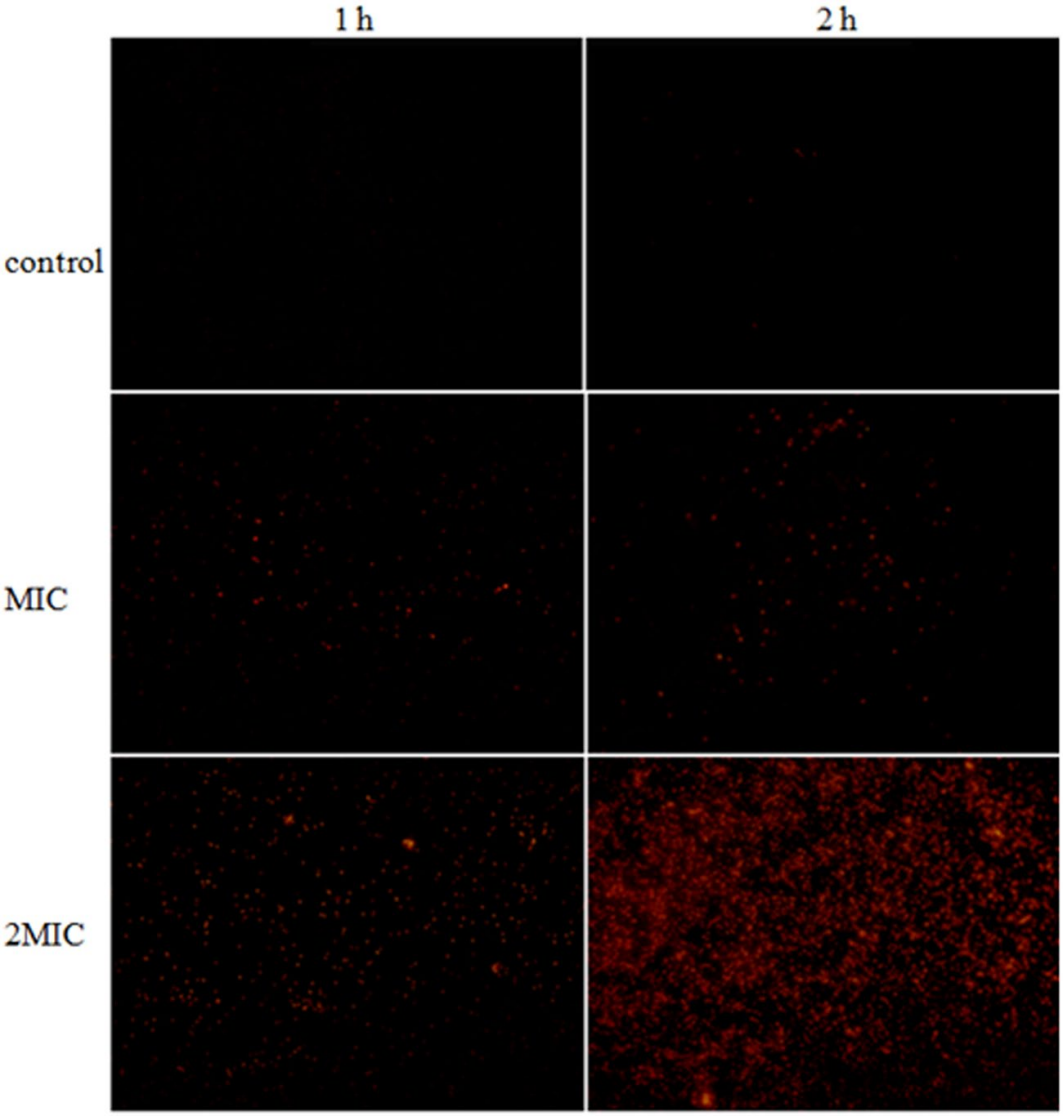
The effect of ME on membrane potential of *S. aureus*.

The effect of ME from hawthorn on intracellular membrane potential was determined by fluorescence inverted microscope probe DiBAC_4_(3). In this process, probe enters the cell, binds to cytoplasmic proteins and emits fluorescence. No fluorescent signal was detected in its unconjugated state, while the cell intracellular membrane potentials increased when the cells were depolarized. As shown in Fig. 4, compared with the control group, the fluorescence intensity significantly increased in a dose- and time-dependent manner when cells were treated with ME.

### Integrity of cell membrane

The integrity of cell membrane was determined by the measurement of the leakage of the intracellular components including reducing sugar, the absorbance at 260 nm and 280 nm of the supernatant of tested bacteria. The results are shown in Table 4. Similarly, the concentration of reducing sugars, nucleic acids and proteins in suspensions treated with ME increased significantly in a dose-dependent manner. These results indicated that bacterial cells exposed to ME suffered the irreversible damage and the integrity of cell membrane was severely destructed.

**Table 4:** The effect of the ME on cell constituents’ release of *S. aureus*.

	Cell constituents’ release		
	OD_260nm_	OD_280nm_	Reducing sugar (µg/mL)
Control	0.027 ± 0.003c	0.053 ± 0.003 c	13.7 ± 1.7 c
MIC	0.298 ± 0.017 b	0.166 ± 0.014 b	46.2 ± 5.2 b
2MIC	0.647 ± 0.031a	0.492 ± 0.025 a	87.3 ± 10.1 a

Values represent means of three independent replicates ± SD. Different letters within a column indicate statistically significant differences (P < 0.05).

### ATPase, SOD and CAT activity assays

**Figure 5:**
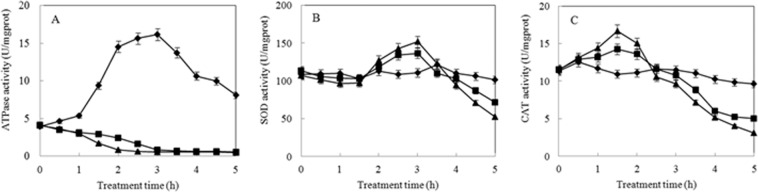
The effect of ME on enzymatic activities. (**A**) ATPase, (**B**) SOD, (**C**) CAT. (♦) control, (▪) MIC and (▴) 2MIC.

The antibacterial role of polyphenols may be partly due to its effect on key enzyme activities of bacterial cells^15^. Key enzymes such as ATPase activity indicates a cell energy metabolism status, while both SOD and CAT activity represents the redox homeostasis. The activity changes of these key enzymes indicate a disturbance of cell homeostasis. As we can see from Fig. 5, the changes of ATPase activity in the control and the ME groups (both MIC and 2MIC) exhibited a completely different trend. The ATPase activity first significantly increased and then decreased in the control while it was significantly blunted in ME treatment. Compared with the control, the activity of SOD and CAT did not change in 2 h and 0.5 h for two treatments, respectively. Hereafter, the activity of SOD and CAT of treatments were first higher and then less than that of the control. After 5 h, SOD and CAT activity decreased by 29.60% and 48.18% in the MIC group while they decreased by 47.97% and 53.47% for the treatment at 2MIC as compared to the control. The reason for the increased SOD and CAT activity could be ME treatment generating ROS that activated SOD and CAT. However, it is likely that the amount of ROS produced by ME treatment were too high to be quenched by SOD and CAT, and these ROS maybe inactivated SOD and CAT, possibly through oxidation. Based on above results, ME treatment could lower effectively the enzyme activity of *S. aureus*, which was reported as an important factor causing bacterial death^15^.

### Production of reactive oxygen species (ROS)

**Figure 6:**
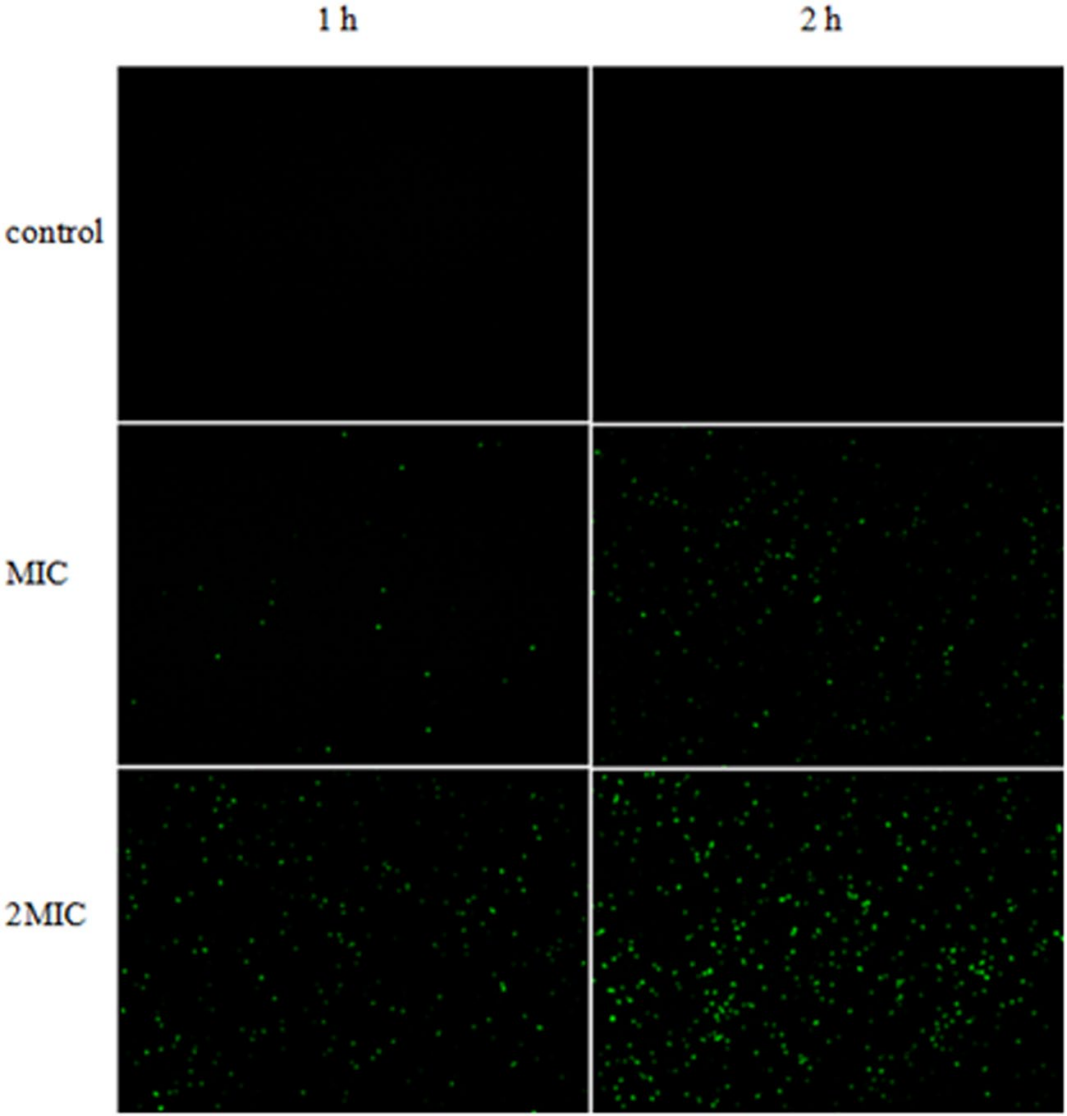
The effect of ME on ROS content in *S. aureus*.

The fluorescent dye 2′,7′ dichlorodihydrofluorescein diacetate (H2-DCFDA) was used to detect ROS, a key factor associated with apoptosis. The results are shown in Fig. 6. It can be seen that the ROS production was increasing in a time- and dose-dependent manner. Only limited ROS produced during the first hour of ME treatment, and ME treatment with a concentration of 2MIC showed a significant increasing green fluorescence intensity. This trend was more obvious after 2 hours of treatment.

### Apoptosis analysis

**Figure 7:**
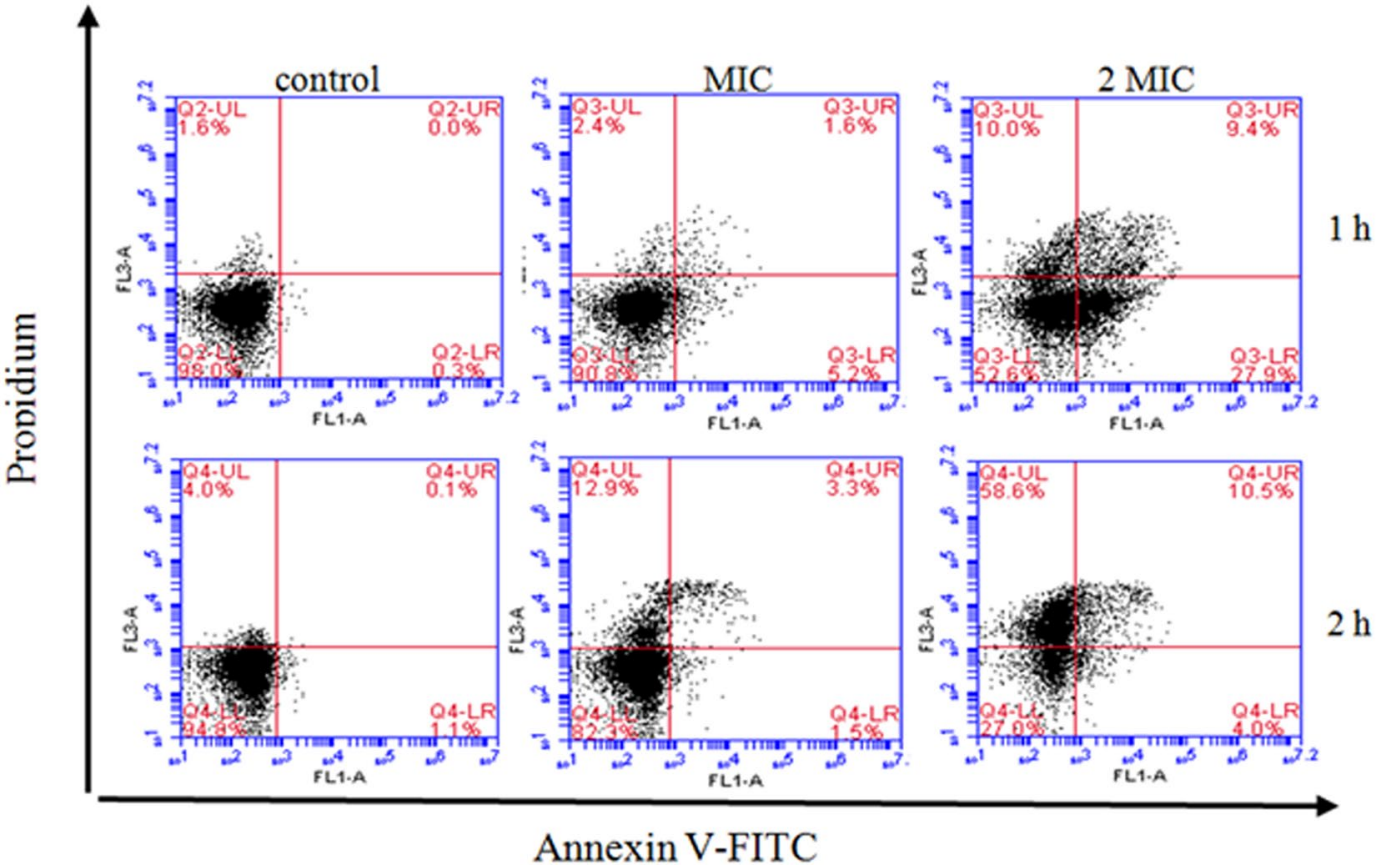
The effect of ME on *S. aureus* cells apoptosis.

Apoptosis-stimulating effects of hawthorn ME against *S. aureus* are shown in Fig. 7. After being incubated for 1 h, the apoptosis rate of the control group was only 1.6%, but the apoptosis rate of *S. aureus* cells significantly increased from 1.6% to 2.4% and 10.0% in two treatments with the MIC and 2MIC of ME, respectively. The apoptotic cell was 0.3% in the control group after incubating 1 h, however, the apoptosis rate significantly increased to 5.2%, 27.9% for early apoptotic cells, and 2.4%, 10.0% for late apoptotic cells after being treated 1 h with MIC, 2 MIC of ME, respectively. After 2 h of treatment, the early apoptosis rate only increased from 1.1% to 1.5% and 4.0% in the MIC and 2MIC group respectively, which was far below that of treatment for 1 h; but the percentage of necrotic cells significantly increased from 4.0% to 12.9% and 58.6% in the MIC and 2MIC groups respectively. These results showed that ME from hawthorn can induce apoptosis, and result in *S. aureus* cells to go into the programmed cell death.

### The changes of gene expressions

**Figure 8:**
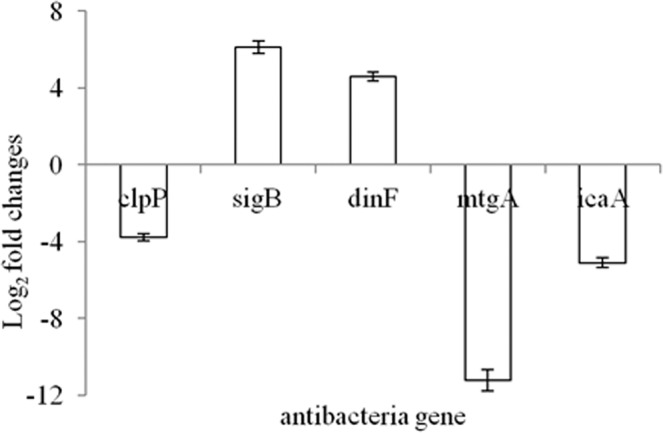
qRT-PCR analysis for expression of antibacterial genes.

The changes of gene expressions related to cell viability (clpP), stress response (sigB), oxidative stress (dinF) and cell wall synthesis (mtgA, icaA) were analyzed by qRT-PCR. The differential expressions of tested genes were observed after treatment with a MIC of ME (Fig. 8). The expressions of clpP and mtgA, icaA were down-regulated, which affected the cell viability and the synthesis of cell wall. The dinF and sigB were significantly up-regulated, which was associated with the homeostatic, oxidative stress or stress response mechanism. When cells are destroyed by oxidative stress, the upregulation of dinF was evidence of cellular survival mechanisms.

### Antibacterial activity in whole milk

**Figure 9:**
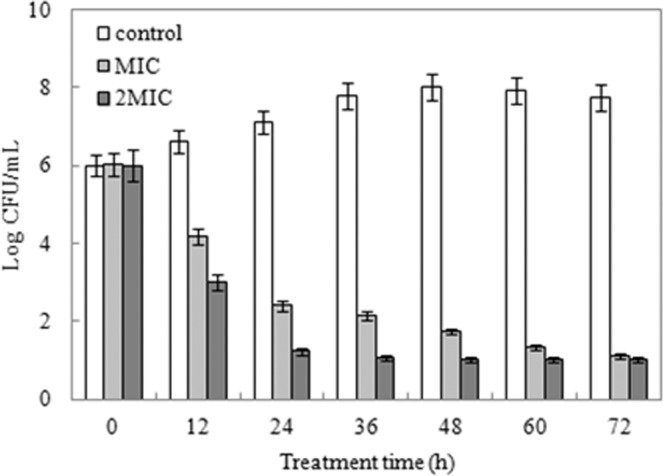
The antimicrobial activity of ME against *S. aureus* in whole milk.

The containment of *S. aureus* were frequently occurred in milk, quick-frozen products as well as various food matrixes and cause severely economic losses and food safety problems^16^. In order to investigate the antibacterial activity of ME from hawthorn in food matrixes, the present study took ultra-high temperature (UHT) whole milk products as an example. The results indicated that ME could effectively reduce the counts of living cells (Fig. 9). Compared to the increase of control, *S. aureus* treated with ME showed a significant decrease in the number of viable cells during cultivation. After 72 h of incubation, the growth of *S. aureus* was inhibited by ME. Treatment at a higher concentration killed the bacteria more efficiently though the number of viable cells in treatments at MIC and 2MIC decreased to the same level after 72 h of treatment.

## Discussion

In this study, the total flavonoids and polyphenols contents of ME from hawthorn were higher than other extracts, and the bacteriostatic efficacy of ME was significantly better than others. The results showed that solvent polarity can have a huge influence on the biological activity of extracts. The solubility of flavonoids and polyphenols can be affected by aromatic ring, glycosyl form and side chain binding. The more hydroxyl groups in the molecule, the stronger of the polarity rises, and easy to dissolve in high polar solvents^17^. The results from the study was similar to previous study that the methanol was most effective in extracting flavonoids and polyphenols from plants^18^. And the research data indicated that hawthorn extracts showed antimicrobial activity, but these results were not entirely consistent^19–21^. These differences may come from different hawthorn species, origins, testing methods as well as different extraction solvents. Stelmakiene *et al*.^22^ also found that hawthorn possessed an inhibitory effect against foodborne pathogens, which supported our results in the present study, indicating that hawthorn extract was a potent bacterial inhibitor. Similar to our findings, other extracts from plants also exhibited inhibitory effects against various foodborne pathogens^23^.

Generally, the total polyphenols played an important role in the antioxidant and antibacterial activities of the extracts, and there was dose-effect relationship between their biological activities and total phenolic content^13,24^. However, for solvent extracts of different polarity, there was not always a one-to-one relationship between the biological activities and the total phenolic content because of differences in the compositions not just the contents of phenolics. Therefore, the antibacterial activities of complex extracts were influenced by its phenolic compounds besides its content. In this study, EAE was rich in polyphenols (almost half of ME), still no inhibition effects for several microorganisms; TE and PE did not exhibit antibacterial activity though they contained a certain total polyphenols, which may be related to the phenolic compositions of extracts.

Some studies reported that the major phenolics of Chinese hawthorn included epicatechin, procyanidin B2, quercetin, hyperoside, isoquercitrin, and chlorogenic acid and so on, among them, the content of epicatechin and procyanidin B2 were much higher than that of other phenolic compounds^25,26^, which was basically similar to the present results. However, there were some differences in the content of some individual phenolic compounds because of differences in extraction methods and experiment methods, as well as origins and varieties of hawthorn. A number of studies have reported that these phenolic compounds are the key bioactive components of hawthorn^6,8,12,26^, but different individual phenolic compounds had different biological activities and levels of activity. Zhang *et al*.^12^ reported the these compounds purified from hawthorn fruits demonstrated varying effect on the oxidation of human low density lipoprotein (LDL) and α-tocopherol activity. Besides, there may be some interactions among phenolic compounds, which can effect their bioactivities by antagonistic or synergistic effects. Iacopini *et al*.^27^ reported that there were antagonistic interactions in all combinations of several compounds including quercetin, catechin, rutin, epicatechin and resveratrol on the DPPH scavenging activity, however, these combinations of the compounds exhibited three different interactions including none, antagonism and synergism in reducing tyrosine nitration. Heo *et al*.^28^ found that there was only an additive effect was observed in combinations of two or three selected phenolics in the experiment of scavenging ABTS radicals. Meyer *et al*.^29^ studied the antioxidant activity of sseveral hydroxyphenols (catechin, quercetin, cyanidin, caffeic acid, and ellagic acid) and combinations of two/three of these phenols by measuring inhibition of human low-density lipoprotein (LDL) oxidation *in vitro*, and concluded that all the antioxidant effects of the hydroxyphenols exhibited additive effects except for combinations including catechin with ellagic acid that played a significant antagonistic role in the antioxidant activity of catechin. The four most abundant polyphenols in ME, including epicatechin, procyanidin B2, chlorogenic acid and quercetin, have been reported to exert antibacterial activities^30–33^. However, contribution of different individual phenolic compounds to the antibacterial activity of the extracts from hawthorn still needs further study.

Two reviews particularly discussed the antibacterial mechanisms of polyphenols and flavonoids^34,35^. They pointed out that antibacterial activities of these compounds were related to various factors, including salt concentration, nutrition availability of pathogens, the cell surface properties of these bacteria as well as the structure of the specific phytochemical compounds such as OH group/groups bonded to both the aromatic rings and the oxygen substituted ring, and number and positions of OH groups. Basically, the interaction between polyphenols and bacteria is non-specific, and depend on the hydrogen group and hydrophobic effects which may have a great effect on lipophilic forces and covalent bond formation^35,36^. So antibacterial activities of many extracts came from different mechanisms. The cell membrane played an important role in the osmotic protection transport processes, biosynthesis of the cell, and its disruption can lead to bacterial death finally^36^. Yi *et al*.^37^ have reported that the tea polyphenols can increase membrane permeability and disrupt cell membranes, resulting in the leakage of cellular molecules. Ren *et al*.^38^ have reported that the pterostilbene derived from Xinjiang wine grape could damage the cell membranes of *S. aureus* and *E. coli*, causing cell membrane depolarization. In this study, the SEM, relative electric conductivity, membrane potential assays indicated that the permeability of bacteria membrane would be increased, and then the contents such as nucleic acids, proteins and other substances within the cell leaked out. As biocatalysts, enzymes were necessary to biological metabolisms and affected by various of factors, and the activity of endoenzyme as one of the main factors can cause the death of bacterial cell^39–41^. Some studies indicated that ROS attack the unsaturated fatty acids in the cell membrane, and cause lipid peroxidation, eventually leading to the destruction of the cell membrane, which can induce the apoptosis of cells^42–44^. Apoptosis was a form of programmed cell death involving the biological regulation of vital activities of cells^45^ and the activation of apoptosis was also considered one of antibacterial mechanisms^46^. Therefore, as a apoptosis factors, the increase of ROS level in treated cells with ME would most likely result in apoptosis of *S. aureus* cells through a membrane-mediated apoptosis pathway combined with the test results of permeability and integrity of cell membrane.

As we all know, clpP, sigB, dinF, mtgA and icaA were genes related to apoptosis^47^. In this study, the expression level of clpP, mtgA and icaA was down-regulated, which indicated that ME inhibited the synthesis of cell wall and membranes. The sigB and dinF related to the biofilm formation, regulating cell homeostasis and stress response mechanism, so obvious up-regulation of their expression level may be related to cell oxidative and environment stress which come from ME from hawthorn. Generally, ME could destroy bacterial cell membranes, and affect the activity of the endoenzyme and the expression levels of genes. Based on these results, we speculate that polyphenols may have a direct interaction with cell membrane. Thereafter, the membrane damages the leakage of intracellular substances. Meanwhile, membrane damages also make the influx various extracellular compounds including polyphenols possible. These stresses results in a great disturbance in intracellular homeostasis, supported by ROS outburst and apoptosis. However, time-dependent comparative analysis should be conducted to finally uncover the exact mechanisms.

A recent study showed that the nisin might interact with phospholipid in milk, which made nisin unable to contact bacteria effectively, and finally reduced its bacteriostatic ability^48^. It was reported that some nutrients including protein and fat in food could combine with antibacterial substances, which resulted in the decrease of antibacterial effect on bacteria^49,50^. The present results showed that the inhibition effect of ME from hawthorn on *S. aureus* has not been affected by these factors, which further confirmed its practical effect as a preservative in whole milk.

## Conclusion

In conclusion, this study indicated that the chemical composition and antibacterial activity of hawthorn extracts were influenced significantly by extracting solvents. Among them, ME and EE from hawthorn showed potent antibacterial activity. Our results suggested that the action mechanism of ME from hawthorn against *S. aureus* might be described as ME from hawthorn causing membrane depolarization and membrane permeabilization, affecting intracellular-enzyme activities and increasing intracellular ROS levels, eventually leading to the cell apoptosis and bacterial death. In addition, ME from hawthorn showed a great antibacterial activity also in food matrix (whole milk), indicating its great potential used in food industry.

## Material and methods

### Plant materials and reagents

Hawthorn cultivar (*C. pinnatifida*) Dajinxing were collected from Linfen, Shandxi Province, China. After harvesting, the seeds were removed and the fruits were dried and stored at −80 °C until the further experiment. Catechin, chlorogenic acid, procyanidin B2, pomolic acid, gallic acid, epicatechin, quercetin, paracoumaric acid, caffeic acid, rutin, hyperoside and isoquercitrin were purchased from Shanghai Yuanye Bio-Technology Co., Ltd. (China). Methanol (HPLC grade) and acetonitrile (HPLC grade) were from Merck (Germany). ATPase Assay Kit, Superoxide Dismutase (SOD) assay kit and CAT Assay Kit were from Jiancheng Bioengineering Institute (China). All other chemicals and reagent used in the experiments were of analytical grade. The standard solutions were prepared by dissolving standards in methanol.

### Microbial strains and culture

Three Gram-positive strains including *Staphylococcus aureus* ATCC 25923, *Bacillus subtilis* ATCC 6051 and *Listeria monocytogenes* ATCC 19115 as well as four Gram-negative strains including *Escherichia coli* ATCC 25922, *Shigella dysenteriae* CMCC (B) 51252, *Salmonella typhimurium* ATCC 19430 and *Pseudomonas aeruginosa* ATCC 9027 were preserved in our laboratory at 4 °C and used in this study.

### Preparation of extracts

Dried, seedless hawthorn fruits were ground with a micro plant grinding machine to a powder. The fruit powder (10.0 g) was extracted three times with 100 mL different solvents (methanol, ethanol, acetone, ethyl acetate, trichloromethane and petroleum ether) and extraction process were continued for 15 min every time. After the removal of solvents with a vacuum rotary evaporator, the extract was dissolved in 10 mL methanol, followed by filtration through a 0.45 μm filter and stored at −40 °C until chemical analyses.

### Determination of total polyphenols

The total polyphenols content was measured by colorimetric Folin–Ciocalteu method^51^. The 0.1 mL of diluted extracts, 2.8 mL of deionized water and 0.1 mL of 1.0 M Folin-Ciocalteu reagent were mixed and stirred. After 8 min, 2 mL of 7.5% sodium carbonate solution was added and mixed thoroughly. The absorbance of the reaction mixtures was measured using a spectrophotometer at 765 nm wavelength after incubation for 2 h at room temperature. Gallic acid was used for calibration of the standard curve and total phenolic content was expressed as milligram gallic acid equivalent per gram dried weight (mg GAE/g DW).

### Determination of total flavonoids

The total flavonoids content was determined following the procedure described by Sultana *et al*.^52^ with slight modifications. Briefly, 1.0 mL of diluted extracts and 0.3 mL of 5% NaNO_2_ solution were mixed for 6 min. Then 0.3 mL 10% Al(NO_3_)_3_ was added and incubated for 6 min. Next, 4 mL of 4% NaOH was added. The final volume was adjusted to 10 mL with distilled water and mixed thoroughly. After 15 min, absorbance of the mixture was determined at 510 nm. Rutin was used for calibration of the standard curve and the content of flavonoids was expressed as milligram rutin equivalent per gram dried weight (mg RE/g DW).

### Antibacterial activity

The *in vitro* antibacterial activity of the tested sample was carried out by Oxford cup method. Briefly, 100 μL of bacterial suspension containing 10^7^ CFU/mL of bacteria were spread on nutrient agar medium. The 100 μL of the sample was loaded on 6 mm sterile Oxford cup. After 24 h of incubation at 37 °C, the diameter of inhibition zone (DIZ) was measured. The minimum inhibitory concentration (MIC) and minimum bactericide concentration (MBC) were determined according to the method as previously described^53^ with minor modifications. Two fold serial dilutions of ME were filtered through 0.22 *μ*m Millipore filters and prepared in sterile NB medium. To each tube, 50 *μ*L of the inoculum containing approximately 1 × 10^6^ CFU/mL microorganisms were added. The tubes were then incubated at 37 °C and examined for evidence of the growth. The MIC was determined as the lowest concentration of the ME that demonstrated no visible growth for incubating for 24 h, while the MBC was the lowest concentration of the test ME that showed no visible growth in the culture incubating at 37 °C for 48 h.

### HPLC analysis

Phenolic composition in the ME were identified and quantified by reverse phase HPLC, using the Agilent 1100 Series (Agilent, USA) separation module equipped with a reversed-phase C_18_ column (250 mm × 4.6 mm, 5.0 μm, Kromasil) according to the method reported by Liu *et al*.^54^. A binary solvent system was employed consisting of formic acid/water (1%, v/v) as solvent A and acetonitrile/methanol (80:20, v/v) as solvent B, and the diode array UV detector (DAD) was set at 280 nm to record the peak intensity. The gradient programme was 0–5 min with 3% solvent B, 5–12 min with 3–10% B, 12–25 min with 10–18% B, 25–35 min with 18–20% B, 35–40 min with 20–80% B, 40–45 min with 80–3% B and 45–50 min with 3% B. The flow rate of the mobile phase was 1.0 mL/min, and the injection volume was 10.0 μL. The column was operated at 30 °C. Identification was performed from the comparison with the retention time of standard, and individual phenolic content was estimated on the basis of peak area and the calibration curves of the corresponding standards.

### Scanning electron microscope (SEM)

SEM observation on the tested bacteria was performed according to the method as described previously^53^ with some modifications. The bacterial cells were incubated in NB at 37 °C for 10 h, and then the suspensions were added 1 × MIC and 2 × MIC of ME. The suspension was incubated at 37 °C for 1 h and 2 h, and then the suspension was centrifuged at 5000 rpm for 10 min. The cells were washed twice with 0.1 M PBS (pH 7.4) and fixed with 2.5% (v/v) glutaraldehyde in 0.1 M PBS overnight at 4 °C. After this, the cells were successively dehydrated using 30%, 50%, 70%, 90%, and 100% ethanol, and then the ethanol was replaced by tertiary butyl alcohol. Then, cells were dried, gold-covered by cathodic spraying. Finally, morphology of the bacterial cell was observed with a scanning electronic microscope (SEM) operated at an accelerating voltage of 25–30 kV.

### Permeability of cell membrane

The permeability of bacteria membrane was expressed in the relative electric conductivity and determined according to the method previously reported^53^. After cultivating, the bacteria were washed with 5% of glucose until their electric conductivities were near to that of 5% glucose, and they were the case for isotonic bacteria. The ME at two different concentrations (MIC, and 2 × MIC) were added to 5% glucose and the isotonic bacteria solution, respectively. After completely mixed, the samples were incubated at 37 °C for 1 h and 2 h. The permeability of bacteria membrane was calculated according to the formula previously reported reported by Diao *et al*.^53^.

### Integrity of cell membrane

The integrity of *S. aureus* strains was determined by measuring the leakage of intracellular components as previously reported^53^ with slight modifications. Cells from the 100 mL working culture of tested *S. aureus* were collected by centrifuged for 10 min at 6000 rpm, washed three times, and resuspended in 0.1 M pH 7.4 phosphate buffer solution. One hundred milliliters of cell suspension were incubated at 37 °C under agitation for 4 h in the presence of ME at three different concentrations (0, MIC and 2 × MIC). Then, 20 mL of samples were collected and centrifuged at 10,000 g for 5 min. And then the concentrations of reducing sugars in supernatant were determined according to the DNS colorimetric reaction, and the absorbance at 575 nm was recorded on a spectrophotometer compared to a glucose calibration curve. In addition, to determine the concentration of the released constituents consisting largely of UV-absorbing compounds such as protein and nucleic acids, 3 mL supernatant were used to measure UV absorption at 280 nm and 260 nm, respectively. Correction was made for the absorption of the suspension with the same PBS containing the same concentration of sample.

### Membrane potential

The effect of ME on membrane potential was evaluated as previously reported with minor exceptions^38^. The bacteria cells were incubated in nutrient broth at 37 °C for 10 h. The suspensions were added MIC and 2MIC of ME, respectively; control culture was left untreated. Next the suspensions were incubated at 37 °C for 1 and 2 h respectively. Before test, membrane potential sensitive fluorescent probe DiBAC_4_(3) was added to a cell suspension with the final concentration of 0.5 μg/mL. After 5 min, the fluorescence intensity was observed under fluorescence inverted microscope (Leica DMi8, Wetzlar, Germany).

### Measurement of cellular adenosine triphosphatase (ATPase), superoxide dismutase (SOD) and Catalase (CAT) activity

The bacterial cells were incubated in NB at 37 °C for 10 h, and then the suspensions were added 1 × MIC and 2 × MIC of ME. After treatment, the extraction of ATPase, SOD and CAT and their activities were determined by the ATPase Assay Kit, SOD assay kit and CAT Assay Kit (Jiancheng Bioengineering Institute, Jiangsu, China) respectively according to the manufacturer’s guidance.

### Determination of reactive oxygen species

The levels of reactive oxygen species (ROS) were measured by the intracellular peroxide-dependent oxidation of 2′,7′- dichlorofluorescein-diacetate (DCFH2-DA) according to the method described by Li *et al*.^42^. The bacteria were cultured in LB medium containing a density of 1 × 10^7^ cells/mL, and then treated with ME (0, MIC and 2MIC) at 37 °C for 30 min. Then the bacteria were collected by centrifugation at 6000 g at room temperature for 10 min, and washed three times in PBS. The fluorescent dye was added and incubated at room temperature for 1 h, then the fluorescence intensities were observed under fluorescence inverted microscope (Leica DMi8, Wetzlar, Germany).

### Apoptosis analysis

Apoptosis analysis assay was carried out according to the methods described by our previous report^55^. The cells containing approximately 1 × 10^8^ CFU/mL were harvested after treated with ME (0, MIC and 2MIC) at 37 °C for 1 and 2 h respectively and stained for 15 min with fluoresced inisothio-cyanate (FITC)-Annexin V and propidium iodide (PI) in the dark at room temperature according to the manufacturer’s recommendations (BD Biosciences). The flow cytometer (FACScan, BD Biosciences) equipped with a CellQuest software (BD Biosciences) was used to analyze 1 × 10^4^ cells. Cells were sorted into living, necrotic, early apoptotic, and late apoptotic cells. The relative ratio of early and late apoptotic cells were counted for further comparison.

### Quantitative real-time polymerase chain reaction (qRT-PCR) analysis

The qRT-PCR analysis was performed according to the method described by Ren *et al*.^38^ with some modifications. The total RNA was extracted from *S. aureus* treated with a MIC of ME and without ME by using TRNzol reagent according to the manufacturer’s instructions. The synthesis of cDNA required 3 μg of RNA using FastQuant RT Kit (TIANGEN, China), following the manufacturer’s protocol. The cDNA was then used as templates for qRT-PCR. The qRT-PCR amplification conditions were as follows: 10 min at 95 °C, 40 cycles of 10 s at 95 °C, 15 s at 50 °C, and 20 s at 72 °C, and data collection was at 60 °C for 15 s. The 16s rRNA housekeeping gene was used as an internal standard to normalize the expression of genes. The primers for clpP, sigB, dinF, mtgA, icaA and 16s rRNA were designed by the free online primer design software Primer3, and were listed in Table 5.

**Table 5:** The primers used in this study.

Primer	Sequence
clpP-F	5′- CACGATCCAGATCCTCTGCC -3′
clpP-R	5′ - GCCCATGTCCAGCGAATAGA - 3′
sigB-F	5′ - AAGTGATTCGTAAGGACGTCT - 3′
sigB-R	5′- TCGATAACTATAACCAAAGCCT - 3′
dinF-F	5′ - GCATCTCGCCTTACCCAT - 3′
dinF-R	5′ - GTTCCAGGACTGCCTCACTA - 3′
mtgA-F	5′ - GCGTTGTTTAGCGTTGC - 3′
mtgA-R	5′ - TCCAGACCGTTTCTATCCC - 3′
icaA-F	5′ - CTGGCGCAGTCAATACTATTTCGGGTGTCT - 3′
icaA-R	5′ - GACCTCCCAATGTTTCTGGAACCAACATCC - 3′
16s rRNA-F	5′ - GGGACCCGCACAAGCGGTGG - 3′
16s rRNA-R	5′ - GGGTTGCGCTCGTTGCGGGA - 3′

### Antibacterial activity in whole milk

*S. aureus* cells were cultured to mid-log phase, collected by centrifugation (6000 g, 10 min), washed thrice in PBS and re-suspended in ultra-high temperature (UHT) whole milk. The final concentration of viability cells in whole milk were up to 10^6^ CFU/mL. Then the milk were treated with MIC, 2MIC of ME respectively. The inoculated samples were incubated at 37 °C for 0, 12, 24, 36, 48, 60 and 72 h. After the designated incubation time, the survivor cells were counted according to the typical colony number of *staphylococcus aureus* on the baird-parker plate.

### Statistical analysis

Statistical analysis was performed on Data Processing System software (DPS, version 7.05). Differences between means were considered statistically significant at *P* < 0.05.

## Supplementary information

Supplementary information.
