# Supplementary material for: Chemical composition, antibacterial activity and action mechanism of different extracts from hawthorn (Crataegus pinnatifida Bge.)
Source: Sci Rep. 2020 Jun 1;10:8876. doi: 10.1038/s41598-020-65802-7 (PMC7264281; doi:10.1038/s41598-020-65802-7)
Supplement: Supplementary file 1 — Supplementary information. [file 41598_2020_65802_MOESM1_ESM.docx]

Chemical composition, antibacterial activity and action mechanism of different extracts from hawthorn (*Crataegus pinnatifida* Bge.)

Liang-Liang Zhang ^1^, Li-Fang Zhang ^1^ and Jian-Guo Xu ^2^**^*^**

^1^ School of Chemistry and Material Science, Shanxi Normal University, Linfen 041004, China; 771677097@qq.com (L-L. Z); sxsd456@sina.com (L-F. Z.);

^2^ School of Food Sciences, Shanxi Normal University, Linfen 041004, China; xjg71@163.com (J-G. X.)

***** Correspondence: xjg71@163.com; Tel.: +86-357-2051247


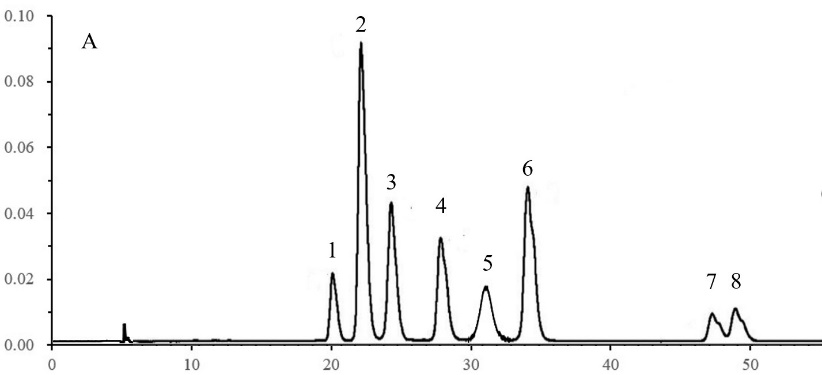


**
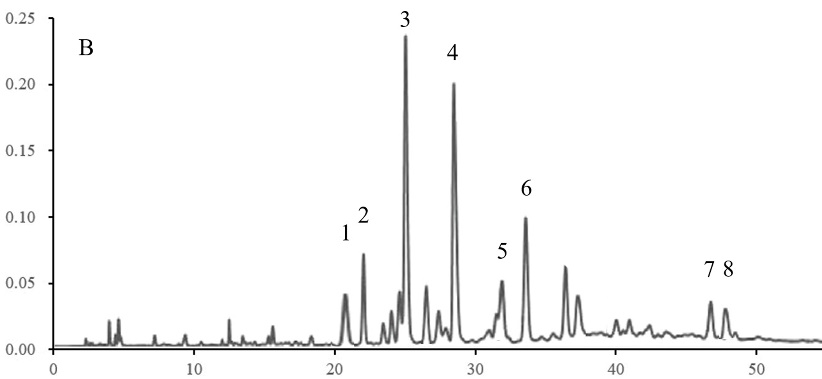
Figure S1.** HPLC chromatograms of reference standards (A) and the ME (B) from hawthorn at wavelength 280 nm. The peaks are (1) catechin, (2) chlorogenic acid, (3) procyanidin B2, (4) epicatechin, (5) quercetin, (6) paracoumaric acid, (7) hyperoside, and (8) isoquercitrin.
